# Deepening the Knowledge of *ROS1* Rearrangements in Non-Small Cell Lung Cancer: Diagnosis, Treatment, Resistance and Concomitant Alterations

**DOI:** 10.3390/ijms222312867

**Published:** 2021-11-28

**Authors:** Giorgia Guaitoli, Federica Bertolini, Stefania Bettelli, Samantha Manfredini, Michela Maur, Lucia Trudu, Beatrice Aramini, Valentina Masciale, Giulia Grisendi, Massimo Dominici, Fausto Barbieri

**Affiliations:** 1 Ph.D. Program Clinical and Experimental Medicine (CEM), Department of Biomedical, Metabolic and Neural Sciences, University of Modena and Reggio Emilia, 41125 Modena, Italy; 2 Oncology and Hematology, Modena University Hospital, University of Modena and Reggio Emilia, 41125 Modena, Italy; lucia.trudu@gmail.com (L.T.); mdominici@unimore.it (M.D.); 3 Oncology and Hematology, Modena University Hospital, 41125 Modena, Italy; bertolini.federica@policlinico.mo.it (F.B.); maur.michela@policlinico.mo.it (M.M.); barbieri.fausto@policlinico.mo.it (F.B.); 4 Molecular Pathology, Modena University Hospital, 41125 Modena, Italy; bettelli.stefania@policlinico.mo.it (S.B.); manfredini.samantha@aou.mo.it (S.M.); 5 Thoracic Surgery Unit, Department of Diagnostic and Specialty Medicine—DIMES of the Alma Mater Studiorum, University of Bologna, G.B. Morgagni—L. Pierantoni Hospital, 47121 Forlì, Italy; beatrice.aramini@auslromagna.it; 6 Laboratory of Cellular Therapy, Program of Cell Therapy and Immuno-Oncology, Division of Oncology, University-Hospital of Modena and Reggio Emilia, Department of Medical and Surgical Sciences for Children & Adults, 41125 Modena, Italy; valentina.masciale@unimore.it (V.M.); giulia.grisendi@unimore.it (G.G.)

**Keywords:** lung cancer, *ROS1* rearrangements, target therapies, molecular alterations, next generation sequencing

## Abstract

*ROS proto-oncogene 1 (ROS1)* rearrangements are reported in about 1–2% of non-squamous non-small-cell lung cancer (NSCLC). After efficacy of crizotinib was demonstrated, identification of *ROS1* translocations in advanced disease became fundamental to give patients the chance of specific and effective treatment. Different methods are available for detection of rearrangements, and probably the real prevalence of *ROS1* rearrangements is higher than that reported in literature, as our capacity to detect gene rearrangements is improving. In particular, with next generation sequencing (NGS) techniques, we are currently able to assess multiple genes simultaneously with increasing sensitivity. This is leading to overcome the “single oncogenic driver” paradigm, and in the very near future, the co-existence of multiple drivers will probably emerge more frequently and represent a therapeutic issue. Since recently, crizotinib has been the only available therapy, but today, many other tyrosine kinase inhibitors (TKI) are emerging and seem promising both in first and subsequent lines of treatment. Indeed, novel inhibitors are also able to overcome resistance mutations to crizotinib, hypothesizing a possible sequential strategy also in *ROS1*-rearranged disease. In this review, we will focus on *ROS1* rearrangements, dealing with diagnostic aspects, new therapeutic options, resistance issues and the coexistence of *ROS1* translocations with other molecular alterations.

## 1. Introduction

Lung cancers are classified into two main histological subtypes: non-small-cell lung cancer (NSCLC) and small-cell lung cancer (SCLC). In the last decade in Western countries, the incidence of SCLC has decreased in favor of NSCLC, which currently represents up to 80–90% of lung cancers (in particular adenocarcinoma histology) [1].

Along with the characterization of NSCLC, according to morphological criteria (mainly adenocarcinoma or squamous cell carcinoma), the assessment of predictive biomarkers and the identification of molecular targets are crucial to define the optimal treatment strategy for advanced disease. 

To date, in clinical practice it is mandatory to test all non-squamous NSCLC for therapy-predictive biomarkers, including targetable oncogenic alterations and immune-oncology biomarkers; in turn, molecular testing is not recommended in squamous cell carcinomas, with the exception of never/light-smokers or long-time ex-smokers [2].

*Epidermal growth factor receptor (EGFR)*-activating mutations are found in 10–20% of Caucasian patients, involving exons 18–21, with exon 19 deletions and L858R substitution in exon 21 being the most common alterations [3]. These mutations are predictive of (variable) sensitivity to EGFR tyrosine kinase inhibitors (TKIs). 

Among adenocarcinomas, 3–7% are characterized by rearrangements involving the *anaplastic lymphoma kinase (ALK)* gene and its fusion partners (usually *EML-4*) on chromosome 2 [4]

More recently, *ROS proto-oncogene 1 (ROS1)* rearrangements have also been identified in about 1–2% of non-squamous NSCLC, and although rare, their identification has become fundamental after target therapies, such as crizotinib, demonstrated robust antitumor activity [5].

*B-type Raf proto-oncogene (BRAF) V600* family mutations have a similar incidence, with V600E being the most common [6]. Clinical trials demonstrated that patients harboring V600E mutation may benefit from combination treatment with BRAF/MEK inhibitors [6]. 

Given the increasing number of treatment options, *EGFR, ALK, ROS1* and *BRAF* testing is now required at the diagnosis of advanced adenocarcinoma to define the best first line treatment. In many laboratories, individual standalone tests are used to test these drivers, but the next-generation sequencing (NGS) technique is being widely adopted to screening oncogenic targets in adenocarcinomas thanks to the capability to provide multiple tests for mutations and fusion genes, even if, for the latter, a confirmatory test by immunohistochemistry (IHC) or fluorescence in situ hybridization (FISH) may sometimes be required [7]. 

Other targetable alterations include RET (Rearranged during Transfection), HER2 (human epidermal growth factor receptor 2), MET (Mesenchymal Epithelial Transition) alterations and NTRK (Neurotrophic Tyrosine Receptor Kinase) 1–3 rearrangements, for which promising targeted therapies are emerging in clinical practice [8,9,10,11,12]. 

Actually, the most common mutations in NSCLC involve *Kirsten rat sarcoma viral oncogene homolog* (*KRAS)* and are reported in about 30% of patients [13,14]. These *KRAS* mutations differ from the above-mentioned alterations since they are usually detected in current/former smokers. Recently, the specific inhibitor sotorasib demonstrated durable clinical benefit in *KRAS* p.G12C-mutated NSCLC [15].

The assessment of programmed death-ligand 1 (PD-L1) expression by IHC assay became mandatory in advanced NSCLC after the approval of pembrolizumab as first line treatment in patients with tumor proportion score (TPS) ≥ 50% or ≥1% in second line [16,17]. PD-L1 expression appears to be marginal in oncogene-addicted disease, where the role of immune checkpoint inhibitors (ICIs) is still debated [18], especially due to the lack of strong prospective evidences and toxicity issues [19,20]. Higher expressions of PD-L1 are more frequent in smokers, while PD-L1 expression ≥ 50% and oncogene alterations rarely overlapped [21].

In this review, we will focus on *ROS1* rearrangements, dealing with diagnostic aspects, new therapeutic options, resistance issues and the coexistence of *ROS1* translocations with other molecular alterations. 

## 2. *ROS1* Biology

ROS1 is a receptor tyrosine kinase belonging to the insulin receptor family. It is composed by an intracellular C-terminal portion containing the kinase domain, a single trans-membrane domain and an extracellular N-terminal domain with multiple fibronectin-type III-like repeats [22]. The *ROS1* gene is located on chromosome 6 (6q22). Very little is known about wild-type *ROS1′*s role, and its ligands were not identified [22] until 2020, when Kiyozumi et al. discovered that neural epidermal growth factor-like 2 (NELL2) binds to the extracellular domain of the mouse ROS1 receptor [23]. Chromosomal rearrangements involving *ROS1* were first identified in glioblastoma cell line U118MG, with the 3′ region of *ROS1* fused to the 5′ region of the FIG gene. *FIG–ROS1* fusions have also been identified in cholangiocarcinoma, gastric cancer and ovarian cancer [24,25]. This fusion promotes tumorigenicity and/or independent growth in different cell lines, even if the mechanism that constitutively activates ROS1 fusion protein is not fully known [26]. Additionally, *ROS1* amplifications have been reported in soft-tissue sarcomas, breast cancer and many other tumor types [27].

The first report of *ROS1* rearrangements in lung cancer dates back to 2007. *ROS1* fusions have been identified on NSLCL line HCC78 (*SLC34A2-ROS1*) and on tumor sample (*CD74-ROS1*), leading to constitutive kinase activation and conferring sensitivity in vitro to TKIs [28]. Since then, several gene fusion partners have been identified, such as *TPM3, SDC4, EZR*, *LRI3, CCD6, SLC34A2* and *TPD52L1* [29]. The *CD74–ROS1* fusion is the most common, accounting for 44% of cases, followed by *EZR–ROS1, SDC4–ROS1* and *SLC34A2* [27].

On ROS1 fusion proteins, the kinase domain is always retained with the junction point at the mRNA level always located on the 5′ end of exons 32–36; in other words, the 3′ region ROS1 kinase domain is fused to the 5′ region of the fusion partner. All fusion genes expressed are oncogenic [30] with ligand-independent, constitutive catalytic activity that activates downstream signaling, including the upregulation of the SHP-2 phosphatase, the MAPK/ERK pathway, the PI3K/AKT/mTOR pathway and the JAK/STAT pathway that regulate cellular survival, growth and proliferation [24,31].

*ROS1*-positive NSCLCs have been identified as a distinct molecular class, such as EGFR or ALK-positive NSCLC [32]. The prevalence of *ROS1* rearrangements in NSCLC ranges from 0.5% to 2%, including East Asian patients [29,30,32,33] and a few cases of squamous cell carcinoma [5,30]. *ROS1* rearrangement in the HCC78 cell line was found to be associated with in vitro sensitivity to crizotinib [32].

*ALK*- and *ROS1*-rearranged tumors are distinct entities, but their kinase domains share common structural characteristics (>80% of the sequence in ATP-binding sites), and this homology justifies the affinity and activity of crizotinib on both kinases, with a half-maximal inhibitory concentration of 40 to 60 nM [34]. Despite these similarities, *ALK* and *ROS1* rearrangements rarely overlap [35].

In vitro models suggest that crizotinib is five times more potent in inhibiting ROS1 than ALK, and this may explain the longer responses achieved in *ROS1*-rearranged NSCLC compared with *ALK*-rearranged ones [5,34].

## 3. Clinicopathological Features

The clinical characteristics of *ROS1*- and *ALK*-rearranged NSCLCs are quite similar: Both rearrangements are more common in younger, never- or light-smoker patients with a histologic diagnosis of adenocarcinoma [36]. Moreover, *ROS1* rearrangements are more common in women [37]. *ROS1* translocations have been associated with the presence of lepidic patterns or extracellular mucin [38]. Disease is commonly diagnosed at an advanced (III or IV) stage [37]. The central nervous system (CNS) is frequently involved in stage IV disease, or CNS may represent the first site of progression on crizotinib given its limited capability to penetrate the blood–brain barrier [39]. Moreover, a higher rate of venous thromboembolism have been reported in *ROS1*-rearranged patients than in unselected NSCLCs patients [40,41].

## 4. *ROS1* Testing Modalities

After the efficacy of crizotinib was demonstrated, the identification of *ROS1* gene rearrangement in advanced NSCLC became fundamental to give patients the chance of specific and effective treatment. Different methods are available for the detection of *ROS1* rearrangements, every technique analyzes products of the different steps of synthesis of rearranged protein, and all have peculiar advantages or limits.

“Break-apart” fluorescence in situ hybridization (FISH) allows the identification of gene rearrangements directly on DNA in interphasic nuclei. As a result of rearrangement, abnormal cDNA or mRNA sequences can be detected by polymerase chain reaction (PCR) or next generation sequencing (NGS). The use of multiplex platforms is able to detect a range of fusion gene transcripts, with the limitation that specific primers are needed: only known fusion variants could be tested with the risk of missing unknown or rare variants, and this may limit its use in clinical practice [26].

As fusion transcript must be translated into protein with tyrosine kinase activity to gain oncogenic function, thus the elevation in protein expression may be interpreted as a surrogate marker for the presence of *ROS1* rearrangement, and this elevation may be detected by immunohistochemistry (IHC) [26]. All these techniques have peculiar advantages and limitations (Table 1), and consensus about the best assay is lacking, but a coordinated use of two or more assays is usually common in real-life laboratories.

The Food and Drug Administration (FDA) approved crizotinib in advance ROS1-positive NSCLC without the requirement of using an approved companion test, and similarly, the European Medicines Agency (EMA) and Italian Medicines Agency (AIFA) just recommend the use of an accurate and validate assay to select patients [42,43].

Although in most laboratories FISH is the gold standard for detection of *ROS1* rearrangements, IHC is an effective screening tool allowing to avoid unnecessary FISH analysis and consequently reduce costs. It is indeed characterized by 100% sensitivity and high specificity (range 92 to 100%). Many experiences have been conducted to compare IHC and FISH assays [44], and different screening modalities have been used across studies to detect *ROS1* rearrangements.

## 5. Immunohistochemistry

Until recently, the only antibody available to test ROS1 expression was D4D6 rabbit monoclonal antibody (Cell Signaling Technology, Danvers, MA, USA) [26]. A globally accepted IHC score system is lacking, as all methods result in very good correlation with FISH [45,46]. Several staining patterns were reported, none of them correlated with specific fusion partners [47]. In the majority of studies, IHC results are reported as 1+ (faint cytoplasmic staining), 2+ (moderate staining) and 3+ (intense staining).

ROS1 IHC can be performed on cytological specimen as well as on formalin-fixed paraffin-embedded (FFPE) tissue specimen or on cellblocks that are often used in lung cancer diagnosis [26]. Positive IHC usually shows a fine and granular cytoplasmatic staining, with the possibility of variable expression levels from cell to cell. Moreover, pathologists should be aware that weak ROS1 expression may be detectable in hyperplastic type II pneumocytes, in alveolar macrophages or in osteoclasts (in case of bone biopsies). This background ROS1 expression makes IHC interpretation less easy than ALK IHC [26]. IHC assay is more accurate in specimens containing ≥20 tumor cells.

Recently, the novel anti-ROS1 antibody SP384 (Ventana Medical System, Tucson, Arizona) was validated as an alternative screening test [48] showing good sensitivity with maintained specificity [49]. 

## 6. Fluorescence In Situ Hybridization

**Figure 1:**
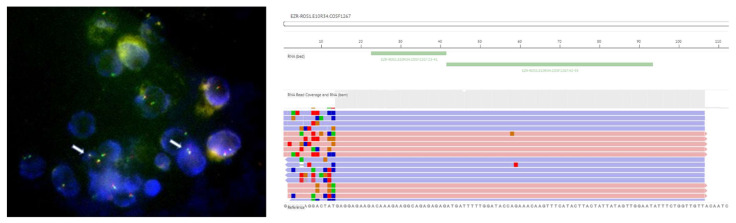
Image of FISH assay detecting *ROS1* rearrangements (indicated by arrows) and image of NGS RNA panel (Oncomine Dx) detecting *EZR–ROS1* fusion. Both tests were performed at our Molecular Pathology Laboratory on cytoblock specimen from pleural effusion in a 55-year-old woman (non-smoker) diagnosed with advanced adenocarcinoma of the lung. In this particular case, IHC screening was positive, but FISH assay was positive for rearrangement only on 8% of cells, not meeting the positivity threshold (≥15%), but fusion was then confirmed by NGS testing. Patient gave her informed consent to publish images and her clinical information.

FISH is considered the gold standard technique in the detection of *ROS1* rearrangements, given its use in clinical trials, including PROFILE 1001 [5]. In this trial, 98% (49/50) of positive patients were tested with FISH assay with split signal in more than 15% of nuclei [5]. Most laboratories use a dual-color break-apart probe design, with two different fluorochromes labelled on the centromeric (3′) and telomeric (5′) parts of the fusion breakpoint. Usually, red and green fluorescent probes are used: when *ROS1* rearrangement is absent, their overlapping produces a “fused” yellow signal, otherwise, red and green signals result separated [26]. *ROS1* positivity may appear with two different patterns: the “classic” one with one fusion signal (native *ROS1*) and two separated 3′ and 5′ signals, or the “atypical” pattern with native *ROS1* fusion signal and an isolated 3′ signal (usually green) without the corresponding 5′ signal (Figure 1) [26,50]. The advantage of FISH is the ability to detect rearrangements without prior knowledge (or hypothesis) of the 5′ fusion partner. 

FISH testing can be performed either on histological sections or on cytological specimens, being aware that tissue sections older than 6 months may report poor hybridization [26]. Criteria for FISH interpretation require evaluating the signals on at least 50 tumor cell nuclei, and the positivity threshold stands at ≥15% [35,51,52].

Experience about multiplex FISH for concomitant detection of *ALK/ROS1* rearrangements on cytological samples has been reported [44].

## 7. Reverse-Transcriptase-Polymerase-Chain-Reaction

Reverse-transcriptase-polymerase-chain-reaction PCR (RT-PCR) allows to identify fusion mRNA and to discriminate among a range of known fusion variants using a multiplex platform. RNA could be extracted from FFPE samples (even if this could affect RNA quality) and RT-PCR is easily performed, rapid and with moderate cost. Notably, RNA integrity could be affected from fixation and processing protocols, and as consequence, the RNA-based PCR failure rate is variable. As already reported, its main limit is that it requires the knowledge of possible fusion partners with “ad hoc” primers, leading to the possibility to miss uncommon or rare variants, despite a growing number of possible *ROS1* fusion partners [26].

## 8. Next Generation Sequencing

NGS technology consists of massive parallel nucleic acids sequencing and allows simultaneous molecular characterization of multiple genes. NGS approaches range from targeted panels that include hotspot regions of variable number of genes to whole exome or whole genome sequencing. Both DNA and RNA (Figure 1) can be used as input material for assays. NGS technology allows the detection of single nucleotide variation, insertion/deletion, copy number variations and genomic rearrangements. Targeted multiplexed panels able to analyze hot-spot regions of all approved molecular biomarker (such as *EGFR, KRAS, BRAF, ALK, ROS1*) are increasingly adopted across molecular pathology laboratories, according to the European Society for Medical Oncology (ESMO) recommendation for NSCLC [53]. 

The advantage of NGS assay for *ROS1* rearrangements is the possibility to detect several fusions and to identify the specific partner of translocation, in addition to the capability of simultaneous analysis of predictive biomarker, saving time and histological material in respect to sequential single-target test.

Some NGS panels are also validated for the molecular analysis of plasma circulating tumor DNA (ctDNA) from liquid biopsies. ctDNA may be released by tumor mass, and its detection may represent a valid tool for early detection, diagnosis or characterization of different tumor types. Indeed, liquid biopsy is gaining increasing relevance in the detection of oncogene alterations in NSCLC, as it may allow overcoming tissue-related issues and to obtain a better sample of tumor heterogeneity in advanced disease or to study resistances during treatments. Several approaches are available to isolate ctDNA and to analyze it, including PCR-based or NGS technologies. To date, the majority of experiences are reporting in *EGFR*-mutated disease, while for *ALK* and *ROS1* rearrangements, this methodology is still under validation [54]. Improvement in the detection of *ROS1* rearrangements on circulating DNA may also lead in the future to the histology-agnostic selection of *ROS1* fusion-positive patients.

## 9. Treatment of *ROS1* Positive Disease

To date, all ROS1 TKIs available are multikinase inhibitors. Early-generation TKIs, such as crizotinib, ceritinib and entrectinib, have demonstrated clinical activity in treatment-naive patients. Next generation TKIs (lorlatinib, repotrectinib and taletrectinib) have shown better intracranial efficacy and activity on resistance mutations following early-generation TKIs. 

In phase I trial, PROFILE 1001 crizotinib showed robust antitumor activity in ROS1 NSCLC, as already demonstrated in *ALK*-rearranged disease [5,55]. The study was designed to include a dose-escalation phase, followed by an expansion phase and was subsequently amended to include an expansion cohort of *ROS1*-rearranged NSCLC. Crizotinib was administered orally at the dose of 250 mg twice a day in continuous 28-day cycles [5]. Fifty-three patients were enrolled in the ROS1 cohort. In all patients except one, *ROS1* rearrangement was detected using break-apart FISH; in the remaining case, RT-PCR was used. All positive FISH had split signal in more than 15% of cell nuclei. Thirty tumor samples were tested with NGS or RT-PCR to identify *ROS1* fusion partners. The most common partner gene was *CD74*, and beyond those already known, two novel partners were also identified: *LIMA1* and *MSN*. Furthermore, 86% of patients had received at least one previous line of therapy for advanced diseases. A 72% ORR was reported, with a median progression free survival (mPFS) of 19.2 months (95% CI 14.4 to not reached) and a median duration of response of 17.6 months (95% CI 14.5 to not reached), both longer than those reported in the ALK cohort [55]. Responses were reported regardless of the *ROS1* fusion partner [5]. The safety profile was similar as in the previous trial [55], with visual-impairment, diarrhea, nausea and peripheral edema as the most common treatment-related adverse events; 94% of adverse events were mild (grade 1 or 2) [5].

The results of crizotinib in *ROS1*-fusion-positive disease were quite relevant, and in 2016, crizotinib was approved by the FDA [56] and subsequently by the EMA for the treatment of patients with advanced *ROS1*-rearranged NSCLC [42]. 

The clinical benefits and safety profile of crizotinib were confirmed in the updated analysis of PROFILE 1001 [57]. After a median follow-up period of 62.6 months, 53 patients received crizotinib, confirming previously reported ORR, which was 72% (95% CI 58% to 83%), and an mPFS of 19.3 months (95% CI 15.2–39.1). Additionally, authors reported 51.4 months (95% CI 29.3—not reached) median overall survival (mOS). Furthermore, 25/53 cases had detectable *ROS1* rearrangements, and 7 different tumor partners were identified; apparently, there was no correlation between different partners and survival, maybe also due to the small number of samples [57].

As in other oncogene-driven diseases, resistance to crizotinib occurred, and at the data cut-off date, death or progression was reported in 46% of patients. Secondary mutation on the tyrosine kinase domain and the activation of an EGFR mediated by-pass pathway were reported as possible mechanisms of resistance [58,59]. 

The retrospective EUROS1 study was conducted in Europe to assess the outcome of ROS1-positive patients treated with crizotinib (standard dose 250 mg twice per day) in a real-world setting. *ROS1* rearrangement was assessed by FISH and was considered positive if at least 15% of tumor cells were rearranged [60]. Thirty-two patients were enrolled and all, except one, were already pretreated for advanced disease. Even with limitations due to the retrospective nature and small sample size, an 80% response rate was reported (higher but consistent with 72% reported in phase I trial) [5,60], while mPFS was 9.1 months, markedly shorter than that reported by Shaw, maybe due to the small sample size, selection bias or the lack of a central validation of ROS1 status [60].

Given the preliminary study that reported sensitivity to pemetrexed in *ROS1*-rearranged tumors [61], the outcome of patients who received pemetrexed (either alone or in combination with platinum) was evaluated. A total of 26 (84%) patients were previously treated with pemetrexed, and their mPFS was 7.2 month with a 57.7% objective response rate (ORR), still with limitations given by the nature of the study [60]. 

After this retrospective report, many phase II trials have been conducted in Europe and East Asia [62,63,64,65]. The results of the main prospective clinical trials with crizotinib are summarized in Table 2. Most of the evidence is concordant with the PROFILE 1001 trial, with the only exception being the AcSé study, which reported only 5.5 months of mPFS (95% CI 4.2–9.1), probably due to a more heavily pretreated population and to poorer performance status (PS) of enrolled patients (25% with ECOG PS 2) [62]. Overall, across studies, crizotinib efficacy did not apparently decrease according to the number of previous treatment lines. 

The OxOnc and EUCROSS trials reported worse outcomes in patients with brain metastases that experienced shorter median PFS when compare with those without brain involvement. These data are to be interpreted with caution, even if they are apparently coherent with limited intracranial efficacy reported with crizotinib when compared with next-generation TKIs in ALK-positive NSCLC (Table 1) [64,65].

Apart from crizotinib, many other TKIs have been investigated in this setting, and their development is awaited, since resistance to crizotinib invariably occurs. 

In vitro studies demonstrated that cabozantinib, a multitargeted TKI, inhibits the survival of ROS1-positive cells, including crizotinib-resistant mutations [66]. In a Korean study, the generation ALK inhibitor ceritinib was tested in *ROS1*-rearranged disease (mostly in treatment-naive patients), reporting outcomes of survival and response similar to PROFILE 1001 [67]. 

Entrectinib (ROS1/NTRK/ALK inhibitor) demonstrated clinical activity as first-line treatment of *ROS1*-rearranged NSCLC, and it is currently the second ROS1 inhibitor achieving FDA and EMA approval based on the pooled analysis of three large multicenter trials (STARTRK-1 and -2 and ALKA-372-001) [68]. Entrectinib showed durable disease control (median DoR 24.6 months; 95% CI 11.4–34.8 and mPFS 19.0 months; 95% CI 12.2–36.6) and relevant intracranial activity (ORR 55%) [68].

Other next-generation TKIs are under investigation both in first and subsequent lines of treatment, given their ability to overcome resistance mechanisms. Indeed, lorlatinib has shown systemic and intracranial activity in patients with *ROS1*-rearranged NSCLC, including patients already pretreated by targeted therapy [69,70], suggesting that is possible to use a sequential approach even in *ROS1*-driven disease as already happens in EGFR and ALK positive NSCLC. In phase I trial by Shaw et al., 69 *ROS1*-positive patients were enrolled, together with ALK-positive ones. The *ROS1* patients were both TKI naive or pretreated with crizotinib, and the ORRs were 64% and 50%, respectively [71]. Among TKI-naive patients with measurable CNS target lesions, 7/11 (64%) had intracranial objective response. The most relevant adverse events reported were hypertriglyceridaemia and hypercholesterolaemia [71].

Other next-generation TKIs, such as repotrectinib [72,73] and taletrectinib [74], have also been studied in TKI-naive patients. Repotrectinib is a next-generation ROS1/TRK inhibitor with great potency against ROS1 receptor [75]; very recently, it achieved FDA Fast Track Designation as a first line treatment of ROS1-fusion-positive NSCLC upon results of a global phase 1/2 trial (NCT03093116, currently recruiting) [75]. In this trial, repotrectinib demonstrated relevant response rates, in particular in the TKI-naive cohort (ORR 86%; 95% CI 42–100) [75]. Of note, clinical and preclinical activity against Gly2032Arg (G2032R) mutation was also reported [76]. 

**Figure 2:**
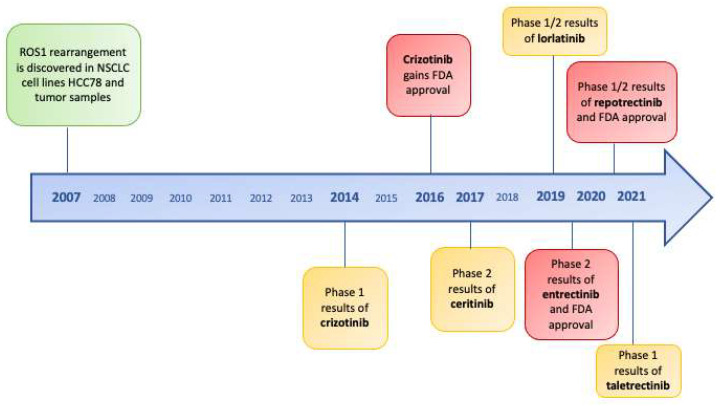
Timeline of ROS1 tirosine kinase inhibitors and FDA approvals since discovery of *ROS1*-rearrangements in non-small-cell lung cancer.

The results of the clinical trials of next-generation inhibitors in TKI-naive patients are summarized in Table 3 and drugs’ development along time is represented in Figure 2.

Lorlatinib, repotrectinib and taletrectinib are not currently approved in Europe. At the failure of approved targeted therapies, *ROS1*-rearranged patients may be address to clinical trials (if available) or to pemetrexed-based chemotherapy given the strong activity of pemetrexed in this subset of patients, which may be explained by low cellular levels of thymidylate synthase [77,78].

Immunotherapy seems to have a marginal role as its efficacy and safety in this subgroup of patients and has not been explored in large series. 

## 10. Resistance

Resistance to crizotinib eventually occurs and mechanisms behind it are not elucidated as well as for other oncogene-driven diseases. The majority (up to 60%) of crizotinib-resistant mutations secondary mutations occur on the kinase domain [58,76], but the activation of bypass signaling pathways (such as EGFR) has also been described [59].

The first documented and predominant mutation responsible of crizotinib resistance is G2032R [79], analogous to *ALK* G1202R. Resistance related to these mutations is not overcome by next-generation inhibitors (as ceritinib, brigatinib and entrectinib in ROS1-positive disease). G2032 is located at the solvent front in the distal end of the kinase hinge; an arginine in this position causes steric hindrance with the piperidine ring of crizotinib, while ATP binding is still possible [58]. G2032R mutation is also able to induce epithelial-mesenchimal transition and to upregulate Twist1, which favors the migratory and invasive capacities of cancer cells [80]. Lorlatinib is not active against G2032R [81], but repotrectinib can target it [76]. 

Additionally, several secondary resistance mutations on the ROS1 tyrosine kinase domain were identified, including D2033, L1951 and the gatekeeper mutation L2026M that may be targetable by lorlatinib or repotrectinib [70]. In the EUCROSS trial, hybrid-capture-based sequencing was performed on the tissue of two patients, revealing *ROS1* L2026M mutation together with TP53 P278H substitution mutation in one case, and PIK3CA E545K substitution in another [64]. Moreover, in the EUCROSS trial, mPFS was significantly longer in TP53 wild-type patients than in TP53-mutant [64].

Other secondary mutations described were L1982F, E1990G and F1994L. On cell lines, the multikinase inhibitor cabozantib inhibits the survival of *CD74-ROS1* cell and of those harboring resistance mutations [82]. 

Additionally, S1986Y/F were described, both conferring resistance to crizotinib and ceritinib but sensitive to lorlatinib in in vitro studies [83].

L2086F substitution is responsible for lorlatinib, crizotinib and entrectinib resistance, although it may be targetable by cabozantinib. 

McCoach et al. performed an analysis of potential resistance mechanisms in a cohort of pretreated *ROS1* and *ALK-*positive NSCLC [84]. In particular, 12 ROS1 patients were included and undergone tumor re-biopsy after radiological progression during treatment with a ROS1 inhibitor including crizotinib, ceritinib and brigatinib. Three patients (25%) had received more than one line of treatment. Ten patients’ samples were analyzed by NGS to sequence exons 36–42: kinase domain mutations were reported in only one patient (p.L2026M and p.L1951R) [84]. On cell lines derived from another patient, resistance to crizotinib was reported through cell proliferation assays, and partial sensitivity was restored by afatinib. In this sample, HER2 was expressed and phosphorylated, but EGFR was not detected [84]. Regarding ROS1-independent mechanisms, mutation in KIT and in β-catenin were detected [84]. A switch from ROS1 to EGFR in survival and control growth signaling pathway has been reported on crizotinib-resistant cell lines, giving preclinical suggestions about the possibility to co-inhibit both targets to prevent resistance [59].

Reciprocally, *ROS1* fusion may emerge as a resistance mechanism to EGFR-TKIs in EGFR-positive NSCLC [85]. 

## 11. *ROS1* Rearrangements and Concomitant Alterations

*ROS1* gene fusion was identified as a distinct molecular class of lung cancer [32,47], such as EGFR and ALK positive cancer. It was commonly assumed that oncogenic mutations mutually exclude each other [29,51], but the recent development of high sensitivity and multiplexed methodologies and their progressive diffusion among diagnostic laboratories has led to the increased detection of concomitant mutation, including targetable or (at least at the moment) non-targetable alterations. 

This subset of multiple-mutated NSCLC currently represents a therapeutic issue, since it is still not clarified if concomitant mutations may have a role in the development of resistance to target therapies or whether they affect tumor microenvironment. Moreover, in case of concomitant “druggable” mutation, it could be difficult to choose the best treatment upfront, and evidence about optimal sequential strategies, depending on the prevalence of molecular pathways, is currently lacking. The coexistence of different mutations may support in the near future the use of combined targeted therapies. 

Many cases of concomitant *ALK/EGFR* mutations have been described, reporting clinical responses to crizotinib [86,87,88]. In addition, the co-expression of EGFR or KRAS in *ALK*-rearranged NSCLC has been reported [89,90]. The role of *KRAS* co-mutations must be clarified, as they were thought to contribute to resistance to EGFR-TKIs [91,92,93], but clinical reports also documented cases of a response to target therapies even in this subgroup [94,95], and this may be related to molecular heterogeneity that characterizes *KRAS*-mutant tumors. 

*ROS1* rearrangements have been also described to co-exists with other oncogenic drivers [45,96,97]. Rimkunas et al. identifies two cases of NSCLC harboring both *ROS1* rearrangement and *EGFR* mutation (L858R and E746-A750 deletion) [47]. E746-A750 deletion concomitant with ROS1 was also reported in a group of 16 ROS1-rearranged samples by Go et al. [98]. A case report described *EGFR, KRAS* and *ROS1* co-mutations in a Chinese patient, harboring clinical benefit with icotinib after experiencing early progression to crizotinib [96].

A Chinese study screened 421 EGFR-positive NSCLC detecting 13 cases (3.1%) with concomitant gene fusions (*ALK* or *ROS1*). Three patients harbored ROS1-rearrangements (two *CD74-ROS1* and one *EZR-ROS1*) and concomitant *EGFR* mutations were L858R in two cases and del19 in one case. When comparing EGFR-positive with “double-positive” patients, the mPFS were 10.7 and 6.6 months, respectively (*p* = 0.004), while no significant difference in OS was reported. Among 13 patients with concomitant alterations, 8 switched to crizotinib at the failure of EGFR-TKI, achieving an mPFS of 6.0 months (95% CI 3.2–8.8) [97].

A retrospective NGS-based analysis on 15 ROS-1 positive lung cancers, 1 case of concurrent MET mutation (R988C), 2 cases with BRAF mutations and 7 cases harboring TP53 mutations were reported: Taken together, all these alterations account for 66.7% of concomitant *ROS1* and other genetic aberrations [99].

Wiesweg et al. in 2016 described a high fraction of ROS1-rearranged patients harboring concomitant mutations [45]. In the study, they screened by IHC 523 patients with advanced or metastatic adenocarcinoma and found 25 (4.8%) ROS1-positive cases. Of these, nine (36%) were found to have concomitant oncogene mutations, including six *EGFR*, two *KRAS* (both patients with smoking history) and one *BRAF* mutation. Of note, all samples were address to FISH analysis and ROS-1 rearrangements (defined with common ≥15% cut-off) were confirmed only in 13 cases (2.5%). Among co-mutated patients, only two *EGFR/ROS1*-positive sample were FISH positive. No correlation was found between IHC staining level and FISH positivity. Additionally, four patients underwent tumor re-biopsies after disease progression, including two patients with *EGFR* and one *KRAS* mutation. These three “double-positive” patients became all FISH positive at tumor re-biopsy; the two EGFR/ROS1 patients had been treated with EGFR-TKIs, and the confirmed FISH positivity at tumor re-biopsy seems to suggest an expansion of the ROS1-positive clone under EGFR target therapy pressure. Five of six *EGFR/ROS1*-positive patients were treated with EGFR TKIs, achieving initial responses that validated the relevance of EGFR positivity, while the role of the accompanying *ROS1* alteration remains unclear. Overall, IHC-positive patients showed better OS when compared with a cohort of EGFR/ALK negative patients. Moreover, the sensitivity to pemetrexed in ROS1-positive patients was also confirmed in this study [45]. The high fraction of patients harboring either EGFR and ROS1 positivity led authors to speculate that ROS1-rearranged and *EGFR*-mutated lung cancer may originate from a common precursor lesion.

Conversely, Lin et al. retrospectively evaluated 62 ROS1-rearranged NSCLC (confirmed by FISH, PCR or sequencing or both techniques), reporting only two cases with concomitant *KRAS* mutation and a higher proportion of concurrent non-druggable mutations, such as *TP53* (25.2%), *CTNNB1* (7%) and *CDKN2A/B* loss (13.6%), whose role and therapeutic relevance must be clarified [100].

In the retrospective EUROS1 study, in one patient, concomitant *ROS1* rearrangement and *KRAS* mutation were reported [60].

NSCLCs are characterized by a high number of somatic mutations [101] and the “single oncogenic driver model” is probably no longer able to describe the emerging variability of oncogene-driven NSCLC. Considering variable clinical behavior and sensitivity to targeted-agents, Skoulidis recently described an “intra-driver heterogeneity” in which co-occurring mutations may have a prominent role [102]. Advanced or metastatic diseases are more likely to harbor concomitant mutations, allowing to speculate that these may take part to tumor progression and metastatic dissemination [102].

The clinical significance of somatic alterations in tumors harboring oncogenic fusions is still not well known. Both *ALK-* and *ROS1*-rearranged tumors present high rates of co-occurring *CDKN2A/2B* mutation and relative lower rates of concurrent *TP53* mutations, although the latter appear to be more common in ROS1-positive tumors than in the *ALK*-rearranged ones, still conserving a negative prognostic role as in *EGFR*-driven tumors [102]. 

## 12. Conclusions

The relevance of the detection of *ROS1* rearrangements in NSCLC is widely recognized. Moreover, the real prevalence of *ROS1* translocations is probably higher than that reported in literature, as our capacity to detect gene rearrangements is improving. In particular, with NGS techniques we are currently able to assess multiple genes simultaneously with increasing sensitivity. The “single oncogenic driver model” is becoming outdated, and in the very near future, the co-existence of multiple drivers will probably emerge more frequently and represent a therapeutic issue. Preclinical and clinical studies should clarify if there are pathways that prevail on others and consequently define new combination strategies.

In this “oncogene-driven” era, although the detection of oncogene targets is often desirable and the possibility to administer targeted agents is appealing, molecular reports should be interpreted with caution, and integration between molecular biologists, pathologists and clinicians is needed with the aim of giving patients the best treatment available. 

The treatment algorithm of *ROS1*-rearranged disease is becoming more and more complex. For many years, crizotinib has been the only available therapy in this setting, but today, new and very promising ROS1 inhibitors are emerging. New generation TKIs show improved penetration across the blood–brain barrier, yielding relevant intracranial response rates or preventing the onset of brain metastases (and relative comorbidities). A direct comparison between TKIs in this setting is still lacking, but novel inhibitors are also able to overcome resistance mutations to crizotinib, hypothesizing a possible sequential TKI strategy also in *ROS1*-rearranged disease. At the failure of target therapies, pemetrexed-based chemotherapy will probably remain a valid option, while the role of immunotherapy in this context is yet to be clarified.

## Figures and Tables

**Table 1:** Advantages and limitations of available diagnostic techniques for *ROS1* rearrangement detection.

	IHC	(RT)-PCR	FISH	NGS
Advantages	Effective screening tool Reduction of costs avoiding unnecessary FISH test Short turnaround time	High specificity and sensitivity Short turnaround time	Low input of material High specificity and sensitivity Short turnaround time Does not require knowledge of possible fusion partners	Simultaneous testing of many predictive biomarkers, saving time and material High specificity and sensitivity Identification of several ROS1 fusion partners Both DNA and RNA as input material Recent validation of panels for ctDNA
Limitations	Lack of globally accepted scores May be difficult to interpret (background ROS1 expression on pneumocytes and alveolar macrophages)	Variable rates of failure (RNA integrity could be affected by fixation) Primers require knowledge of possible fusion partners Missing of uncommon or rare partners	Difficult to interpret (expertise of pathologist is needed) ROS1 fusion partners are not specified	Longer turnaround time Reduced sensitivity of DNA-based assays in detection of rearrangements Possible RNA failure in RNA-based assays

**Table 2:** Main prospective clinical trials with crizotinib.

Clinical Trial	Phase	N of Patients	Median Age (Range)	ROS1 Testing Techinique	Previous Lines	ORR% (95% CI)	mPFS Months (95% CI)	mOS Months (95% CI)	CNS Outcomes
PROFILE 1001 [57]	1	53	53 (25–77)	51 FISH 2 RT-PCR	≥0	72 (58–83)	19.3 (15.2–39.1)	51.4 (29.3–NR)	-
OxOnc [65]	2	127	51.5 (22.8–79.7)	RT-PCR	≤3	71.7 (63.0–79.3)	15.9 (12.9–24)	32.5 (32.5–NR)	mPFS 10.2 (95% CI 5.6–13.1) vs. 18.8 months (13.1–NR) ^a^
EUCROSS [64]	2	34 ^b^	56 (33–84)	FISH ^c^	16 ≤ 1 14 ≥ 2	70 (51–85)	20.0 (10.1–NR)	NR (17.1–NR)	mPFS 9.4 (1.7–NR) vs. 20.0 months (10.1–NR) HR 1.53; 95% CI 0.488–4.7; *p* = 0.464) ^a^
AcSè [62]	2	37 ^d^	62 (33–81)	FISH	median 2 (range 1–7)	69.4 (53–82) ^e^	5.5 (4.2–9.1)	17.2 (6.8–32.8)	-
METROS [63]	2	26	68 (28–86)	FISH	≥1	65 (44–82)	22.8 (15.2–30.3)	NR	ORR 33% (2/6)

ORR: Objective Response Rate; mPFS: median Progression Free Survival; mOS: median Overall Survival; CNS: Central Nervous System; FISH: Fluorescense in situ Hybridization; RT-PCR: Reverse-transcriptase-polymerase-chain-reaction; NR: Not Reached; HR: Hazard Ratio. ^a^ Patients with baseline brain metastases versus patients without brain metastases; ^b^ 4 patients were excluded from efficacy analysis; ^c^ DNA Sequencing on 20 samples, with confirmed rearrangements on 18 samples; ^d^ 36 evaluable; ^e^ best overall response rate; ORR assessed at two cycles 47.2% (95% CI 30.4–64.5).

**Table 3:** Main clinical trials about next generation TKIs as first-line treatment.

Drug	Phase	Number of ROS1 TKI-Naive Patients	ROS1 Testing Technique	ORR % (95% CI)	mPFS (95% CI)	Intracranial Activity
Entrectinib [68]	½ ^a^	53	FISH, PCR, NGS	77 (64–88)	19.0 (12.2–36.6)	RR 55% (32–77)
Ceritinib [67]	2	30	FISH	62 ^b^ (45–77)	19.3 (1–37)	DCR 63% (31–86)
Lorlatinib [71]	1/2	21	FISH, PCR, NGS	62 (38–82)	-	RR 64% (31–89)
Repotrectinib [72]	1/2	7	NR	86 (42–100)	-	-
Taletrectinib [74]	1	11	FISH, PCR, NGS	66.7 (34.5–87.9)	29.1 (2.6–NR)	-

TKI: Tirosine Kinase Inhibitor; ORR: Objective Response Rate; mPFS: median Progression Free Survival; RR: Response Rate; DCR: Disease Control Rate; NR: Not Reached. ^a^ integrated analysis of 3 trials; ^b^ 28 patients evaluable for response.
